# Myeloid-Specific Acly Deletion Alters Macrophage Phenotype *In Vitro* and *In Vivo* without Affecting Tumor Growth

**DOI:** 10.3390/cancers13123054

**Published:** 2021-06-19

**Authors:** Kyra E. de Goede, Sanne G. S. Verberk, Jeroen Baardman, Karl J. Harber, Yvette van Kooyk, Menno P. J. de Winther, Sjoerd T. T. Schetters, Jan Van den Bossche

**Affiliations:** 1 Department of Molecular Cell Biology and Immunology, Cancer Center Amsterdam, Amsterdam Gastroenterology Endocrinology Metabolism, Amsterdam UMC, Vrije Universiteit Amsterdam, 1081 HZ Amsterdam, The Netherlands; k.degoede@amsterdamumc.nl (K.E.d.G.); s.verberk@amsterdamumc.nl (S.G.S.V.); k.harber@amsterdamumc.nl (K.J.H.); y.vankooyk@amsterdamumc.nl (Y.v.K.); sjoerds@irc.vib-ugent.be (S.T.T.S.); 2 Department of Medical Biochemistry, Experimental Vascular Biology, Amsterdam Cardiovascular Sciences, Amsterdam UMC, University of Amsterdam, 1105 AZ Amsterdam, The Netherlands; j.baardman@amsterdamumc.nl (J.B.); m.dewinther@amsterdamumc.nl (M.P.J.d.W.)

**Keywords:** ATP citrate lyase, macrophage, tumor-associated macrophage, immunometabolism, cancer

## Abstract

**Simple Summary:**

Tumor cells show different metabolism compared to normal tissue. For instance, they increase ATP-citrate lyase (Acly) expression for increased proliferation. Acly is therefore currently being targeted for cancer therapy. However, targeting this enzyme may have side effects on other cells within the tumor microenvironment, including tumor-associated macrophages (TAMs) which are immune cells crucial for cancer progression. As Acly deletion previously showed increased pro-inflammatory responses of macrophages *in vitro*, we hypothesized that Acly-deficient TAMs may better control tumor growth. Here, we determined the effect of Acly deficiency specifically in pro-cancer anti-inflammatory macrophages and TAMs in two mouse models of cancer. While Acly is crucial for anti-inflammatory macrophage activation, Acly-deficient TAMs do not affect tumor growth. Together, our results indicate that targeting Acly as anti-cancer therapy has no adverse effects on TAMs.

**Abstract:**

Cancer cells rely on ATP-citrate lyase (Acly)-derived acetyl-CoA for lipid biogenesis and proliferation, marking Acly as a promising therapeutic target. However, inhibitors may have side effects on tumor-associated macrophages (TAMs). TAMs are innate immune cells abundant in the tumor microenvironment (TME) and play central roles in tumorigenesis, progression and therapy response. Since macrophage Acly deletion was previously shown to elicit macrophages with increased pro- and decreased anti-inflammatory responses *in vitro*, we hypothesized that Acly targeting may elicit anti-tumor responses in macrophages, whilst inhibiting cancer cell proliferation. Here, we used a myeloid-specific knockout model to validate that absence of Acly decreases IL-4-induced macrophage activation. Using two distinct tumor models, we demonstrate that Acly deletion slightly alters tumor immune composition and TAM phenotype in a tumor type-dependent manner without affecting tumor growth. Together, our results indicate that targeting Acly in macrophages does not have detrimental effects on myeloid cells.

## 1. Introduction

In order to sustain proliferation, cancer cells exhibit altered metabolism compared to untransformed cells. One example of this is the activation of ATP-citrate lyase (Acly), a metabolic enzyme converting citrate to acetyl-CoA. Acly is often overexpressed in cancers and correlates with worse prognosis [1,2]. Acly-derived acetyl-CoA was previously found to be important for tumor growth and proliferation, as it is the first building block for lipid biogenesis [3,4]. Furthermore, acetyl-CoA affects gene transcription programs by altering histone acetylation in cancer cells [5,6]. Therefore, Acly has been suggested as a promising therapeutic target for cancer [4,5,6,7,8]. However, a challenge in targeting cancer metabolism is posed by potential side effects on the metabolism of other stromal cells present within the tumor microenvironment (TME), including tumor-associated macrophages (TAMs). TAMs are innate immune cells abundant in the TME that often show a pro-tumoral polarization, contributing to tumor growth, metastasis and therapy resistance [9]. 

Intracellular metabolism is increasingly being recognized as a crucial regulator of immune cell function [10,11,12,13,14]. In LPS-activated macrophages (M(LPS)), increased glycolysis and anabolic pathways like the pentose phosphate pathway and fatty acid synthesis support inflammation and immune effector functions, whilst enhanced mitochondrial oxidative phosphorylation and fatty acid oxidation (FAO) characterize IL-4-activated macrophages (M(IL-4)) [12,14]. Both *in vitro* LPS and IL-4 stimulation activate Acly to increase acetyl-CoA levels, leading to histone acetylation, chromatin opening and induction of gene transcription [15,16]. However, care should be taken when interpreting these studies using pharmacological inhibitors. Indeed, such compounds often show off-target effects as shown for etomoxir [17]. Previous studies with this carnitine palmitoyl-transferase 1 (CPT1) inhibitor led to the common belief that FAO supports M(IL-4) macrophages [18]. A decade later, experiments with CPT1- and CPT2-deficient macrophages challenge this dogma and demonstrate that FAO is mostly unnecessary for IL-4-induced macrophage responses [19,20]. This aforementioned experience with etomoxir highlights the urgent need to validate earlier observations with Acly inhibitors using genetic knockout models. This became even more important since a newer study reported that silencing ACLY in human macrophages does not affect the IL-4-induced expression of a selected set of genes [21]. Moreover, Acly inhibitors still suppress IL-4-responses in human ACLY knockdown cells [21], questioning previous pharmacological approaches and the importance of Acly in mouse IL-4-activated macrophages. 

To investigate the role of Acly in macrophages, we recently created a myeloid-specific Acly knockout mouse model (Acly^M-KO^) [22]. Acly^M-KO^ bone marrow-derived macrophages (BMDMs) show increased production of inflammatory mediators in response to LPS stimulation and downregulate specific IL-4-induced markers *in vitro* [22]. These results indicate that Acly deletion induces a pro-inflammatory macrophage phenotype *in vitro* which may potentially translate to an anti-tumor phenotype *in vivo*. This reprogramming of TAMs has been proposed as a strategy to dampen tumor growth [23,24,25,26]. While increased inflammation in the tumor microenvironment is not always beneficial [9,27], pro-inflammatory macrophages can present tumor-associated antigens and activate effector T cells [28,29]. Therefore, we hypothesized that Acly inhibition might be beneficial in combatting tumor growth both by direct inhibition of cancer cell proliferation as previously demonstrated [3,4,5,6] and by reprogramming macrophages towards an anti-tumor phenotype.

Here, we show in a myeloid-specific knockout (KO) model in an unbiased manner that loss of Acly diminishes IL-4-responses and that the altered phenotype of Acly^M-KO^ TAMs does not affect tumor growth. Our results may contribute to the investigation of cancer therapies and suggest that Acly inhibition aimed to inhibit cancer cell proliferation will likely not pose negative impacts on TAMs. 

## 2. Materials and Methods

### 2.1. Mice and Bone Marrow-Derived Macrophage Culture

Mice with a myeloid-specific Acly deletion were created by crossing C57Bl/6J mice with loxP sites around exon 9 of the Acly gene (Acly^fl/fl^) [30] with Lyz2-Cre transgenic mice. All mouse experiments were approved by the Committee for Animal Welfare (University of Amsterdam and VU University Amsterdam). Acly^fl/fl^ and Acly^M-KO^ mice were sacrificed and femurs and tibias were flushed to collect bone marrow cells. Bone marrow precursors were subsequently differentiated to macrophages using RPMI-1640 (Gibco, Waltham, MA, USA) containing 25 mM HEPES (Gibco), 2 mM L-glutamine (Lonza, Basel, Switzerland), 10% FCS (Biowest, Nuaillé, France), 100 U/mL penicillin (Lonza), 100 μg/mL streptomycin (Lonza) and 15% L929-conditioned medium for 7 days. Macrophages were stimulated for 24 h with 100 U/mL recombinant murine IL-4 (PeproTech, Rocky Hill, NJ, USA) or left untreated as controls.

### 2.2. Tumor Models

Mouse MC38 colon carcinoma cells were cultured in DMEM (Gibco) supplemented with 10% FCS, 100 U/mL penicillin and 100 μg/mL streptomycin and mouse 3LLR lewis lung carcinoma cells [31,32] were cultured in RPMI 1640 containing 10% FCS, 100 U/mL penicillin, 100 μg/mL streptomycin and 2 mM L-glutamine prior to injection. Then, 8-16-week-old mice of similar male/female distribution per genotype were anesthetized using 3% isofluorane, and tumor cells in serum-free medium were injected subcutaneously. MC38 cells were injected at 200,000 cells/mouse in 50 μL while 3LLR cells were injected at 1 million cells/mouse in 200 μL. Tumors were measured 3 times per week using a digital caliper in a blinded manner by the same person. Tumor volume was calculated as 1/6 × π × x^3^ (x = average of length and width of tumor). Mice were sacrificed when tumors became visibly necrotic, reached a tumor size > 2000 mm^3^, or at the endpoint of the study (21 days for MC38, 12 days for 3LLR). At study endpoint, tumors and spleens were removed (for MC38, *n* = 13 for Ctrl and *n* = 11 for Acly^M-KO^; for 3LLR, *n* = 12 for Ctrl and *n* = 11 for Acly^M-KO^). 

### 2.3. Sample Preparation and Antibody Staining

Sample preparation, antibody staining and analysis were performed as previously reported [33]. Tumors and spleens were cut into pieces with scissors in the presence of 385 μg/mL liberaseTL enzyme mix (Merck, Darmstadt, Germany) and incubated for 25 min at 37 °C on a shaker. After resuspending, ice-cold RPMI 1640 containing 10% FCS, 10 mM EDTA (Merck), 20 mM HEPES and 50 μM β-mercaptoethanol (Gibco) was added to inactivate digestion enzymes for 10 min at 4 °C while shaking. Cells were filtered over a 70 μm cell strainer and washed extensively. Spleens were additionally subjected to ACK lysis buffer (Gibco) for 3 min at room temperature. Cells were subsequently plated as 3 × 10^6^ cells/well in a 96 V-bottom plate. 

For antibody staining, cells were washed in freshly made phosphate buffered saline (PBS)/bovine serum albumin (BSA) 0.5% and stained for cell surface markers for 30 min in the dark at 4 °C in the presence of anti-CD16/CD32 Fc block (eBioscience, San Diego, CA, USA). After staining, cells were washed twice with PBS and fixed in 2% PFA for 15 min at room temperature, washed with PBS/BSA and transferred to a FACS plate. For intracellular iNOS and Arg1 staining, cells were permeabilized using 0.1% saponin (Riedel de Haen, Charlotte, NC, USA) in PBS/BSA 0.5% for 15 min at room temperature and stained with antibodies during 30 min in the dark at 4 °C. Cells were subsequently washed with PBS/BSA 0.5% and transferred to a FACS plate. For each tissue, fluorescence minus one (FMO) controls were created by pooling cells of control and Acly^M-KO^ groups and stained using the same protocol. 

A complete list of antibodies used can be found in Supplementary Table S1. 

### 2.4. Flow Cytometry and Analysis

All samples were acquired at the O|2 Flow Facility at Amsterdam UMC (Netherlands) on an X20 Fortessa flow cytometer (BD Biosciences, Franklin Lakes, NJ, USA) which was calibrated daily using CS&T calibration beads (BD Biosciences). Cells were filtered using a 70 μm cell strainer before measuring and acquired using a plate loader set to 1.0 μL/s acquisition speed. Appropriate single stain and FMO controls were measured on the same day as the samples. 

Data were analyzed using FlowJo (TreeStar, Ashland, OR, USA, v10) and were first compensated using single stains with UltraComp eBeads (ThermoFisher, Waltham, MA, USA) labeled with the appropriate fluorochrome. Compensation was subsequently verified using FMO controls. Next, cells were gated on FSC-A/SSC-A to gate out debris, then on viable CD45^+^ cells and lastly on FSC-A/FSC-H to identify single cells. Further gating was performed as indicated in the text. 

For unbiased tSNE analyses, files were uploaded to the OMIQ online analysis platform (https://omiq.ai/, accessed on 8 March 2021) and gating was reproduced to subsample cells. In OMIQ, the tSNE tool was used to create tSNE clustering. For analysis of immune cell composition, single live CD45^+^ cells were gated, while for analysis of TAM marker expression, single live CD45^+^CD11b^hi^Ly6G^−^CD64^+^ cells were included. For both applications, a maximum of 50,000 single cells was included per sample and 1500 iterations with a perplexity of 30 and a theta of 0.5 were performed. 

Manual gating was executed as represented by tSNE and overlaid on tSNE clustering. For quantification, this manual gating was recreated in FlowJo and the appropriate tissue- and subset-specific FMO median fluorescent intensity (MFI) was subtracted from MFI of phenotypic markers. Graphs were visualized and statistical analyses were performed in GraphPad Prism (San Diego, CA, USA, V.8.2.1). Corrected MFI was plotted in heatmaps using the package pheatmap in an R environment calculating a row z-score. 

### 2.5. Transcriptomics

RNA-sequencing and analysis were performed as previously described [34]. In short, the RNeasy Mini Kit with DNase treatment (QIAGEN, Hilden, Germany) was used to isolate total RNA from BMDMs. RNA was used to construct strand-specific libraries with the KAPA mRNA Hyper-Prep kit (KAPA Biosystems, Wilmington, MA, USA). Samples were sequenced on an Illumina HiSeq 4000 instrument (Illumina, San Diego, CA, USA) and reads were aligned to the mouse genome mm10 by STAR 2.5.2b with default settings. Differential expression was analyzed using the R package DESeq2 and volcano plots and heatmaps were generated using the ggplot2, ggrepel and pheatmap packages. Pathway analysis was performed using the Metascape analysis platform (https://metascape.org/, accessed on 31 January 2021) [35] using upregulated genes between IL-4-treated Acly Control and Acly^M-KO^ BMDMs.2.6. Statistical analysis

Data are reported as mean ± standard error of the mean (SEM) of *n* = 3 biological replicates for transcriptomics. Sample size was estimated based on similar assays in our previous publications and calculated as *n* = 13 per group for *in vivo* experiments including potential losses due to humane endpoints. Statistical significance was evaluated using a two-tailed Student’s *t*-test or two-way ANOVA followed by Sidak’s post hoc multiple comparison test in GraphPad Prism software. Outliers were identified by ROUT test with *Q* = 1% and removed from analyses. *p* values < 0.05 were considered significant, with * *p* < 0.05, ** *p* < 0.01, *** *p* < 0.001.

## 3. Results

### 3.1. Transcriptomics Analysis of Myeloid-Specific Acly-Deficient Mice Validates the Involvement of Acly in IL-4-Induced Macrophage Activation

**Figure 1:**
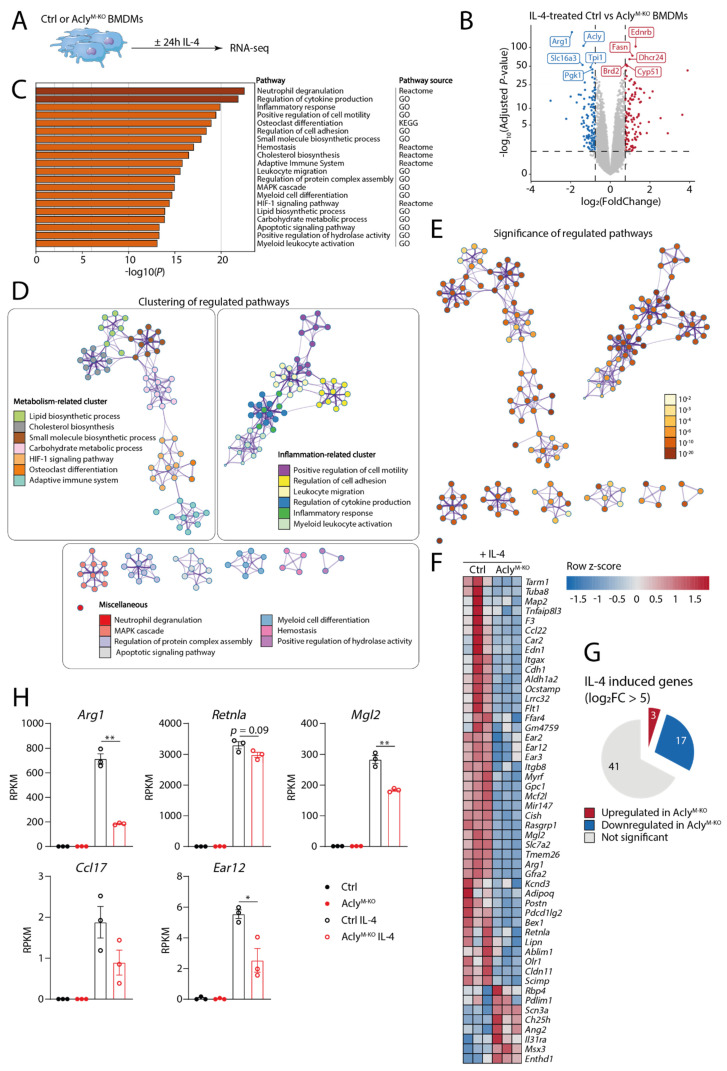
Acly regulates IL-4-induced macrophage activation *in vitro*. (**A**) Acly^fl/fl^ Ctrl and Acly^M-KO^ BMDMs were left untreated or were treated with IL-4 for 24 h for RNA sequencing. (**B**) Volcano plot of IL-4-treated Acly^fl/fl^ Ctrl and Acly^M-KO^ BMDMs. (**C**) Top 20 differentially regulated pathways of IL-4-treated Acly^fl/fl^ Ctrl and Acly^M-KO^ BMDMs. (**D**) Networks with clustered pathways as identified in (**C**), colored by cluster and (**E**) by *p*-value. (**F**) Heatmap showing the effect of Acly^M-KO^ on top 50 highly IL-4-induced genes. (**G**) Pie chart quantifying up-, down-, and not significantly regulated genes (*p* adjusted < 0.05, log_2_FC > |0.58|) by Acly^M-KO^ on highly IL-4-induced genes. (**H**) Expression of genes previously identified as impacted by Acly inhibition for control and Acly^M-KO^ BMDMs, naive or treated with IL-4. *p*-values are indicated as <0.05 *, <0.01 **. Results are shown as mean ± SEM from *n* = 3 technical replicates from three pooled mice.

Earlier studies with Acly inhibitors and siRNAs indicate that Acly activity and downstream acetyl-CoA production are required for IL-4-induced macrophage activation [15]. Additionally, we reported earlier that the IL-4-induced expression of at least a selection of genes and surface markers was reduced in myeloid-specific Acly-deficient mice (Acly^M-KO^) [22]. To follow up on these observations in a more comprehensive and unbiased manner, we performed RNA-sequencing on naive and IL-4-treated BMDMs derived from Acly^M-KO^ and Acly^fl/fl^ littermate controls (Ctrl) (Figure 1A). Together with the prototypical IL-4-induced gene Arg1, Acly was among the most significantly downregulated genes in Acly^M-KO^ macrophages. This confirmed the efficiency of Acly knockdown in our model (Figure 1B). Subsequent pathway analysis indicated effects of Acly deficiency on pathways related to inflammation, motility, migration and adhesion, and metabolism (Figure 1C). As many pathways are related and genes may overlap between pathways, we visualized the top-20 most regulated pathways in clustered networks. These further confirmed the two main and highly significantly regulated clusters related to inflammation and metabolism (Figure 1D,E). Genes within these pathways did not show clear directionality regarding up- or downregulation (Supplementary Figure S1A–C). 

To specifically assess the effect of Acly deficiency on IL-4 responses in macrophages, we first selected the top IL-4-induced genes in Ctrl BMDMs (log2FoldChange > 5 and adjusted *p*-value < 0.05). Validating our approach, these included common IL-4-induced marker genes including *Arg1*, *Cdh1*, *Cldn11*, *Ear12* and *Mgl2* (Figure 1F) [36,37]. Of the selected 61 highly IL-4-induced genes, 17 were downregulated and only 3 genes were upregulated in IL-4-treated Acly^M-KO^ compared to IL-4-treated Ctrl BMDMs (Figure 1G). Only two of these 20 significantly regulated genes could be found within differentially expressed genes in unstimulated conditions with neither achieving significance (Supplementary Figure S1D), indicating that Acly effects on IL-4-induced genes are not present on baseline.

While previous studies already indicated that short-term Acly inhibition or siRNA-mediated knockdown decreases IL-4 signaling [15], earlier experiences with the Cpt1 inhibitor etomoxir indicate that inhibitor studies need to be interpreted with caution [17,19]. To further validate the importance of Acly in regulating IL-4-induced macrophage responses, we specifically assessed the effect of myeloid Acly deficiency on the expression of typical IL-4-induced marker genes that were shown to be suppressed by pharmacological Acly inhibition. Confirming earlier inhibitor studies, data obtained in Acly^M-KO^ BMDMs highlight the need for Acly for the IL-4-induced induction for most of those marker genes (Figure 1F–H). Together, our unbiased analysis of the Acly^M-KO^ genetic mouse model that we recently generated confirms the importance of Acly for IL-4-induced macrophage responses.

### 3.2. Acly^M-KO^ Macrophages Do Not Improve In Vivo Anti-Tumor Responses

**Figure 2:**
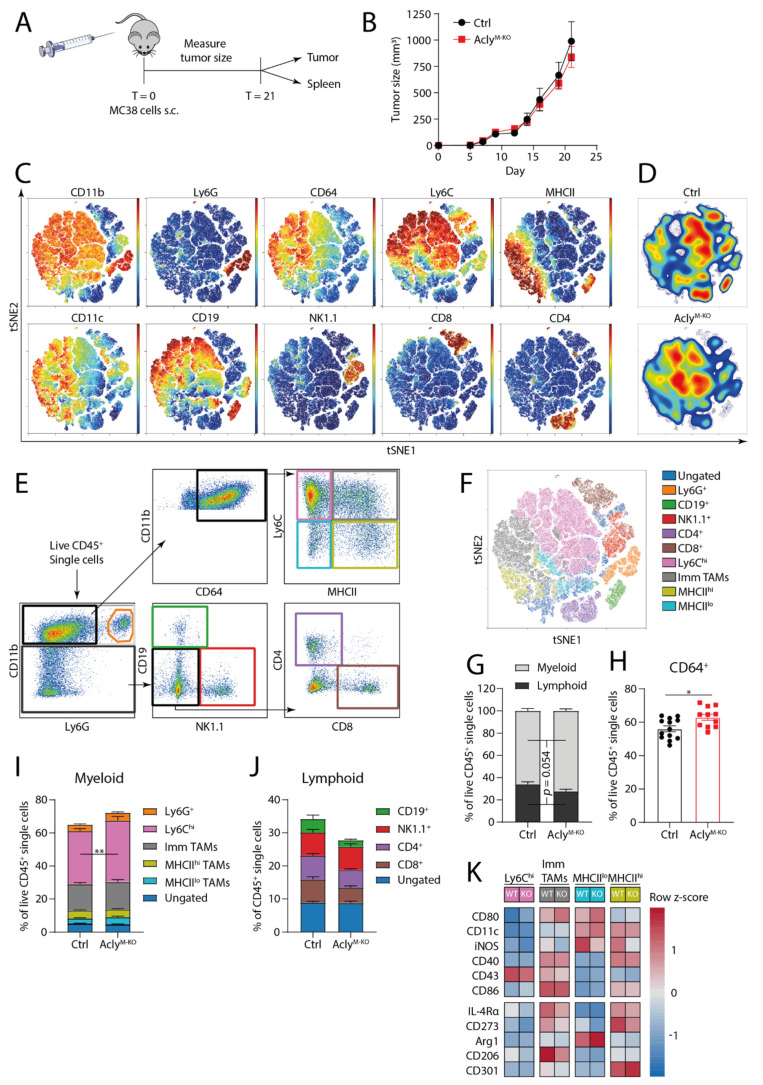
Acly^M-KO^ macrophages do not improve *in vivo* anti-tumor responses in an MC38 tumor model. (**A**) MC38 colon carcinomas were subcutaneously injected and grown for 21 days. (**B**) Growth curves of MC38 tumors for Acly^fl/fl^ Ctrl and Acly^M-KO^ mice. (**C**) Unsupervised clustering analysis of live CD45^+^ single cells isolated from MC38 tumors. (**D**) Density distribution for control and Acly^M-KO^ mice over the unsupervised clustering. (**E**) Manual gating strategy of different immune cell populations. (**F**) Overlay of manually gated populations from (**E**) on unsupervised clustering in (**C**). (**G**) Percentages of CD11b^+^ or CD11b^−^ cells of live CD45^+^ single cells. (**H**) Percentage of CD64^+^ of live CD45^+^ single cells. (**I**) Percentages of myeloid or (**J**) lymphoid cell populations live CD45^+^ single cells. (**K**) Heatmap of pro- and anti-inflammatory macrophage marker protein expression in Ly6C^hi^, Immature TAM, MHCII^lo^ and MHCII^hi^ TAM populations as measured by FACS. For F-K, colors match the colors used for manual gating in (**E**). *p*-values are indicated as <0.05 *, <0.01 **. Results are shown as mean ± SEM.

Reverting tumor-promoting into anti-tumoral TAMs has been proposed as a strategy to dampen tumor growth [23,24,25]. Since our observation above and earlier data indicate that Acly-deficient macrophages display a potentially anti-tumoral phenotype [22], we next questioned whether Acly^M-KO^ mice would better control tumor growth and employed two distinct tumor models to investigate this. In the first model, MC38 colon carcinoma cells were injected subcutaneously (s.c.) and tumors were grown for 21 days (Figure 2A). We measured tumor size over time and observed that myeloid Acly deletion did not affect tumor progression (Figure 2B). As Acly^M-KO^ can still modulate immune cell composition or macrophage phenotype without affecting tumor growth, we investigated immune cell abundance by multiplex flow cytometry and unsupervised clustering (Figure 2C). tSNE analysis distinguished clear clusters of Ly6G^+^, CD19^+^, NK1.1^+^, CD8^+^ and CD4^+^ cells. Additionally, a large myeloid cluster consisting of CD11b^+^CD64^+^ cells showed differential expression of Ly6C and MHCII within the cluster (Figure 2C). Density distributions indicated a decrease in the CD11b^−^ lymphoid clusters and an increased presence of myeloid CD64^+^ and Ly6C^hi^ cells in the absence of Acly (Figure 2D). Manual gating showed considerable overlap with the clusters identified by tSNE analysis (Figure 2E,F) and confirmed the increased relative abundance of myeloid cells over lymphoid cells in tumors of Acly^M-KO^ mice (Figure 2G). Especially CD64^+^ and Ly6C^hi^ myeloid cells showed a higher abundance in MC38 tumors of Acly^M-KO^ mice (Figure 2H,I). Within the lymphoid compartment of MC38 tumors, CD19^+^, CD4^+^ and CD8^+^ T cell subsets were less abundant in the Acly^M-KO^ group but did not reach statistical significance (Figure 2J). No systemic differences in immune composition were found (Supplementary Figure S2A–D). 

As we observed that Acly-deficiency affects macrophage phenotypes, we further investigated the expression of phenotypic markers on different CD11b^hi^/CD64^+^ TAM subsets as gated based on distinct MHCII/Ly6C expression as shown in Figure 2E. While the expression of IL-4-induced surface markers CD206 and CD273 appeared to be reduced in certain TAM subsets of Acly^M-KO^ mice, other markers associated with pro-tumoral TAMs like arginase-1 and CD301 expression tended to be increased. Conversely, the expression of CD80, iNOS and CD11c inflammation-associated proteins seemed higher in the absence of Acly. Yet, none of these differences reached statistical significance (Figure 2K). In summary, while myeloid-specific Acly-deficiency modulated the relative abundance of different myeloid and lymphoid immune cell subsets, this did not translate into a significantly altered TAM phenotype and had no effect on tumor growth in the MC38 tumor model. 

**Figure 3:**
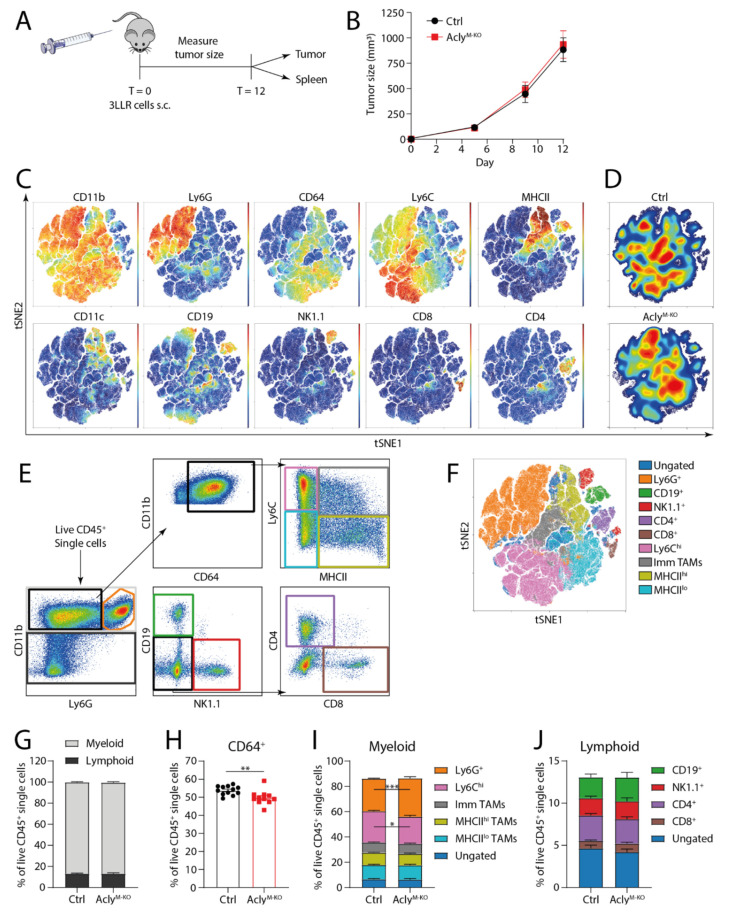
Acly^M-KO^ macrophages do not improve *in vivo* anti-tumor responses in a 3LLR tumor model. (**A**) 3LLR lewis lung carcinomas were subcutaneously grown for 12 days. (**B**) Growth curves of 3LLR tumors for Acly^fl/fl^ and Acly^M-KO^ mice. (**C**) Unsupervised clustering analysis of live CD45^+^ single cells isolated from 3LLR tumors. (**D**) Density distribution for control and Acly^M-KO^ mice over the unsupervised clustering. (**E**) Manual gating strategy of different immune cell populations. (**F**) Overlay of manually gated populations from (**E**) on unsupervised clustering in (**C**). (**G**) Percentages of CD11b^+^ or CD11b^−^ cells of live CD45^+^ single cells. (**H**) Percentage of CD64^+^ of live CD45^+^ single cells. (**I**) Percentages of myeloid or (**J**) lymphoid cell populations live CD45^+^ single cells. For F–J, colors match the colors used for manual gating in (**E**). *p*-values are indicated as <0.05 *, <0.01 **, <0.001 ***. Results are shown as mean ± SEM.

Since effects of myeloid Acly deletion may be tumor type-specific, we also investigated the effects of myeloid Acly deletion in a Lewis lung carcinoma model (3LLR). Subcutaneously injected tumor cells showed no difference in tumor expansion between control and Acly^M-KO^ mice in this model (Figure 3A,B). Similar to the MC38 model, unsupervised clustering identified distinct Ly6G^+^, CD19^+^, NK1.1^+^, CD4^+^ and CD8^+^ subpopulations. The CD11b^hi^CD64^+^ myeloid cell cluster displayed a clear gradient of MHCII and Ly6C expression (Figure 3C). Density distributions in this tumor model indicated an increased presence of Ly6G^+^ and reduced abundance of Ly6C^hi^ cells in tumors of Acly^M-KO^ mice (Figure 3D) and this was confirmed by manual gating (Figure 3E,F,I). While CD64^+^ cells were less abundant in 3LLR tumor-bearing Acly^M-KO^ mice, quantification of the total myeloid/lymphoid distribution and the lymphoid compartment revealed no differences between both groups (Figure 3G–J). No systemic differences in immune composition were found (Supplementary Figure S2E–H). Together, myeloid Acly deficiency slightly altered myeloid subset abundances in 3LLR tumors but this did not translate into altered tumor growth.

### 3.3. Acly Modulates In Vivo TAM Phenotype in 3LLR Tumors

**Figure 4:**
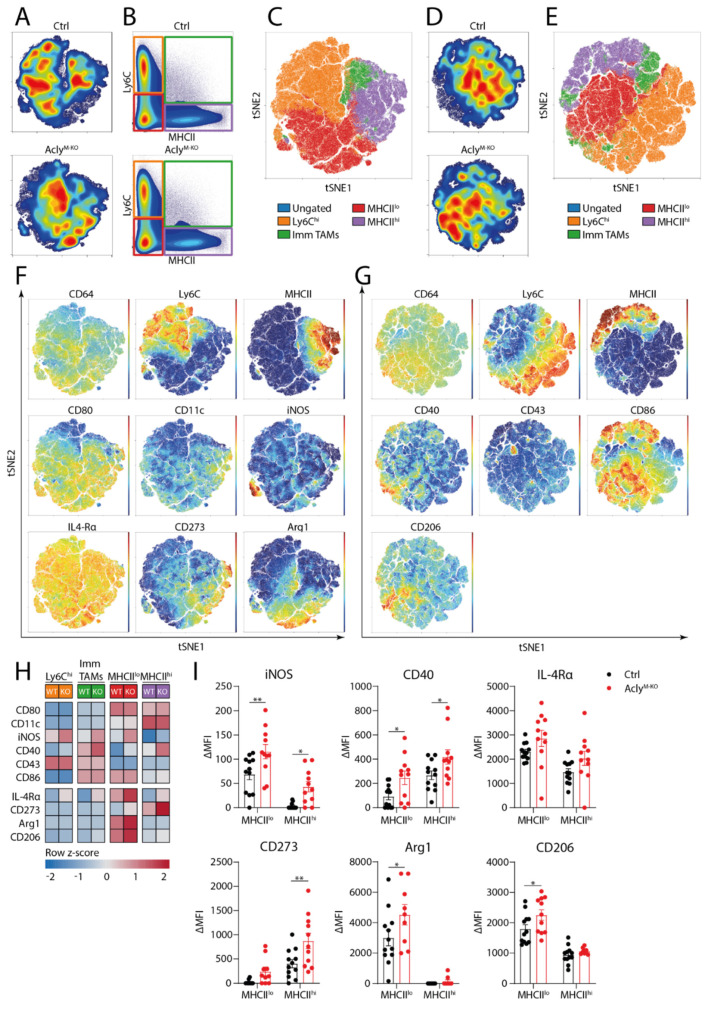
Acly^M-KO^ modulates *in vivo* TAM phenotype in a 3LLR tumor model. (**A**,**D**) Density distributions of control and Acly^M-KO^ mice over unsupervised clustering plots of CD11b^+^Ly6G^−^CD64^+^ myeloid cells. (**B**) Manual gating of MHCII/Ly6C subsets and (**C**,**E**) overlays of these subsets on tSNE clustering. (**F**,**G**) Unsupervised clustering of CD11b^+^Ly6G^−^CD64^+^ cells according to MHCII/Ly6C expression and expression of selected pro- and anti-inflammatory macrophage markers. (**H**) Heatmap of Ly6C^hi^, Immature TAMs, MHCII^lo^ and MHCII^hi^ TAM subset macrophage marker expression and (**I**) ΔMFI of selected macrophage markers for MHCII^lo^ and MHCII^hi^ TAM subsets of control and Acly^M-KO^ mice. ∆MFI was calculated as MFI(sample)-MFI(FMO) on the same subset. *P*-values are indicated as <0.05 *, <0.01 **. Results are shown as mean ± SEM.

Besides immune cell composition, we also investigated surface marker expression on TAM subsets in 3LLR tumors of Acly^M-KO^ and littermate controls. To gain an unbiased overview of *in vivo* TAM phenotype, we performed unsupervised clustering analysis on gated CD11b^hi^Ly6G^−^CD64^+^ macrophages. Two separate flow cytometry panels were designed containing the same CD64/MHCII/Ly6C backbone but different macrophage markers. Density plots of tSNE clustering, together with manual gating of monocyte and TAM subsets, showed small but non-significant shifts from Ly6C^hi^ monocytes towards immature and MHCII^lo^ TAMs (Figure 4A–E). Myeloid subpopulations showed the same trends as presented in Figure 3I (Supplementary Figure S3A,B). In general, Ly6C^−^MHCII^hi^ TAMs had high expression of CD11c, CD40 and CD273 and low levels of iNOS (Figure 4F,G). Other markers such as CD80, IL-4Rα and CD86 showed uniform expression across the TAM subsets, while arginase-1 and CD206 were the highest on Ly6C^−^MHCII^lo^ TAMs (Figure 4F,G). 

When comparing differences in marker expression on TAM subsets from both genotypes, we observed increased iNOS, CD40 and CD273 expression on both MHCII^hi^ and MHCII^lo^ TAM subsets from Acly^M-KO^ mice (Figure 4H,I). Moreover, MHCII^lo^ TAMs showed higher levels of arginase-1 and CD206 in Acly^M-KO^ mice (Figure 4H,I). While this does not indicate a phenotypic shift towards either anti- or pro-tumorigenic TAMs, these results are consistent with the more complex *in vivo* phenotype of macrophages compared to the conventional pro/anti-inflammatory spectrum *in vitro*. To summarize, these data suggest that myeloid Acly deletion increases both pro- and anti-inflammatory TAM phenotypic marker expression in an *in vivo* 3LLR tumor model. 

## 4. Discussion

In the present paper, we validated the importance of Acly for IL-4-induced macrophage activation with a myeloid-specific KO model in which Acly loss diminished IL-4-induced responses. The importance of Acly for M(IL-4) activation was demonstrated by differential regulation of inflammation- and metabolism-related gene expression clusters, illustrating the bridge formed by Acly between metabolism and macrophage function. In Acly-deficient macrophages, most common IL-4-induced marker genes were downregulated, including genes identified previously as being downregulated by Acly inhibitors [15]. Hereby, our genetic approach using Acly-deficient macrophages confirms earlier findings with inhibitors and univocally shows that Acly regulates IL-4 responses in mouse macrophages [15]. Upon IL-4 activation, Akt/mTORC1 signaling was shown to induce Acly activity by phosphorylation, thereby increasing acetyl-CoA production [15]. Acetyl-CoA subsequently increases histone acetylation, allowing gene transcription of some, but not all, IL-4-induced genes [15]. Gene-specific histone acetylation by Acly-derived acetyl-CoA has also been shown during LPS stimulation [5,16,38]. However, the exact mechanisms by which Acly-derived acetyl-CoA allows specific transcription of different gene sets for LPS and IL-4-activation remain to be elucidated. 

It was previously shown that Acly deletion downregulates the IL-4 response of macrophages [22]. We here confirmed these results in an unbiased manner. Generally, Acly supports proliferation by providing acetyl-CoA for fatty acid synthesis and histone acetylation and inhibition of Acly activity was previously found to decrease cell cycle progression [39]. Consistently, in an earlier report, we found “cell cycle checkpoints” as one of the top differentially regulated pathways in Acly^M-KO^ BMDMs [22]. In line with these results, cholesterol and lipid biosynthesis, apoptotic signaling and myeloid cell differentiation pathways remained top differentially regulated pathways even after IL-4-activation of BMDMs, indicating the importance of Acly for the regulation of these pathways. In summary, the genetic approach applied here unambiguously demonstrates the involvement of Acly in mouse IL-4-induced macrophage activation and shows that previous conclusions based on Acly inhibitors in mouse macrophages still hold up. However, the importance of Acly for IL-4-induced macrophage responses may be subject to species differences [21] which need to be elucidated further. 

As Acly-deficient macrophages were previously shown to polarize towards an anti-tumor phenotype *in vitro* [22], we next assessed the ability of Acly^M-KO^ mice to elicit anti-tumor responses in two distinct tumor models. Acly^M-KO^ mice slightly altered the immune composition within the TME and phenotypically altered TAM subsets in the 3LLR model without affecting tumor growth. The effect of myeloid Acly-deficiency on tumor immune composition was tumor type-specific, as lymphoid cell subsets were slightly decreased in the MC38 but unaffected in the 3LLR model. Likewise, while the CD64^+^ and Ly6C^hi^ populations were increased in abundance in MC38-bearing Acly^M-KO^ mice, both populations were decreased in the 3LLR model. Conversely, an increase in Ly6G^+^ neutrophils was found in 3LLR which was not present in MC38. Abundance changes in these populations in either direction did not affect tumor growth in both models. Furthermore, phenotypic markers only showed trends towards upregulation of pro-inflammatory markers in the MHCII^lo^ population and decreases of anti-inflammatory markers in both MHCII^hi^ and MHCII^lo^ TAMs in MC38-bearing Acly^M-KO^ mice. In contrast, Acly^M-KO^ MHCII^lo^ and MHCII^hi^ TAMs upregulated both pro- and anti-tumoral phenotypic markers in 3LLR. This did not indicate clear directionality of TAM polarization towards either a pro- or anti-tumoral phenotype in the absence of Acly. However, none of the changes observed were sufficiently large to affect tumor growth. Our data are consistent with earlier reports of a 3LLR model in which gene expression for iNOS, IL-4Rα and CD206 was decreased in MHCII^hi^ compared to MHCII^lo^ TAMs [32]. In a sarcoma model, CD206^+^IL-4Rα^+^CD64^+^ macrophages were mainly found in progressively growing tumors and were reduced by immune checkpoint therapy, whereas iNOS^+^ macrophages were not found early on but were induced upon immune therapy [40]. The latter effect was most likely caused by IFNγ production of rejuvenated T cells [40]. Our findings of low iNOS expression even in the MHCII^hi^ TAMs may be explained by the fact that we did not employ immune checkpoint blockade and therefore have no elevated IFNγ production by T cells. The question remains whether Acly^M-KO^ could enhance the effects of immune checkpoint therapy in skewing TAMs towards an anti-tumor phenotype. 

The present study illustrates that macrophage profiling according to the typical pro/anti-inflammatory markers and readouts *in vitro* is not predictive for what happens inside the TME where macrophages are exposed to a complex mixture of stimuli with potentially opposing effects. An earlier study activating macrophages with a variety of stimuli has extended the traditional dichotomous pro/anti-inflammatory *in vitro* model to a spectrum with at least nine different polarization states [41]. TAMs created by a cytokine cocktail differ from both M(LPS) and M(IL-4) macrophages [42] and co-express pro- and anti-inflammatory markers but eventually progress towards a pro-tumor phenotype [43]. Moreover, TAM subsets might also acquire an anti-inflammatory profile independent of IL-4 as was previously illustrated for wound healing macrophages [44]. As such, it is difficult to directly translate *in vitro* observations to the *in vivo* environment.

Furthermore, co-expression of pro- and anti-inflammatory markers on TAMs was also found in breast cancer and glioma patients [45,46]. LPS/IFNγ and IL-4 stimulation further negatively cross-regulate transcriptional programs and specific gene expression differs on a single-cell level [43,47], indicating the difficulty to predict functional consequences of TAM polarization *in vivo*. *In vivo* macrophage responses in cancer remain to be fully elucidated and likely differ regarding spatiotemporal characteristics and between monocyte-derived and tissue-resident macrophages [46,48]. The latter may further explain why no major differences were found *in vivo*: the presented tumor models highly depend on tumor-infiltrating monocytes which are highly plastic due to low epigenetic rigidity [49]. Therefore, Acly-mediated metabolic changes may only start playing a role with more differentiated (tumor-associated) macrophages, which are more transcriptionally rigid and may depend more on intracellular regulation of epigenetics. This may be especially important since the applied tumor models are quite aggressive and might therefore not allow revealing potential anti-tumor effects associated with Acly targeting.

## 5. Conclusions

In conclusion, Acly is crucial for IL-4-induced mouse macrophage activation, and myeloid-specific KO of Acly slightly affects tumor immune composition and TAM phenotype without affecting tumor growth, suggesting that cancer therapies targeting Acly do not negatively affect TAMs.

## Data Availability

The accession number for the RNA sequencing data reported in this paper is GSE169187.

## Supplementary Materials

The following are available online at https://www.mdpi.com/article/10.3390/cancers13123054/s1, Figure S1: Acly^M-KO^ BMDMs do not show directionality in their IL-4-induced pathways and IL-4-induced genes are not affected at baseline, Figure S2: Acly^M-KO^ does not affect splenic immune composition during *in vivo* tumor models, Figure S3: Validation of myeloid subpopulation distribution in macrophage marker panels of 3LLR tumor model. Table S1: List of antibodies used.

## References

[1] Wen J., Min X., Shen M., Hua Q., Han Y., Zhao L., Liu L., Huang G., Liu J., Zhao X. (2019). ACLY facilitates colon cancer cell metastasis by CTNNB1. J. Exp. Clin. Cancer Res..

[2] Chen Y., Li K., Gong D., Zhang J., Li Q., Zhao G., Lin P. (2020). ACLY: A biomarker of recurrence in breast cancer. Pathol Res. Pract..

[3] Bauer D.E., Hatzivassiliou G., Zhao F., Andreadis C., Thompson C.B. (2005). ATP citrate lyase is an important component of cell growth and transformation. Oncogene.

[4] Hatzivassiliou G., Zhao F., Bauer D.E., Andreadis C., Shaw A.N., Dhanak D., Hingorani S.R., Tuveson D.A., Thompson C.B. (2005). ATP citrate lyase inhibition can suppress tumor cell growth. Cancer Cell.

[5] Wellen K.E., Hatzivassiliou G., Sachdeva U.M., Bui T.V., Cross J.R., Thompson C.B. (2009). ATP-citrate lyase links cellular metabolism to histone acetylation. Science.

[6] Carrer A., Trefely S., Zhao S., Campbell S.L., Norgard R.J., Schultz K.C., Sidoli S., Parris J.L.D., Affronti H.C., Sivanand S. (2019). Acetyl-coa metabolism supports multistep pancreatic tumorigenesis. Cancer Discov..

[7] Shah S., Carriveau W.J., Li J., Campbell S.L., Kopinski P.K., Lim H.W., Daurio N., Trefely S., Won K.J., Wallace D.C. (2016). Targeting ACLY sensitizes castration-resistant prostate cancer cells to AR antagonism by impinging on an ACLY-AMPK-AR feedback mechanism. Oncotarget.

[8] Khwairakpam A.D., Shyamananda M.S., Sailo B.L., Rathnakaram S.R., Padmavathi G., Kotoky J., Kunnumakkara A.B. (2015). ATP citrate lyase (ACLY): A promising target for cancer prevention and treatment. Curr. Drug Targets.

[9] Greten F.R., Grivennikov S.I. (2019). Inflammation and cancer: Triggers, mechanisms, and consequences. Immunity.

[10] De Goede K.E., Harber K.J., Van den Bossche J. (2019). Let’s enter the wonderful world of immunometabolites. Trends Endocrinol. Metab..

[11] Van den Bossche J., Baardman J., Otto N.A., van der Velden S., Neele A.E., van den Berg S.M., Luque-Martin R., Chen H.J., Boshuizen M.C., Ahmed M. (2016). Mitochondrial Dysfunction Prevents Repolarization of Inflammatory Macrophages. Cell Rep..

[12] Jha A.K., Huang S.C., Sergushichev A., Lampropoulou V., Ivanova Y., Loginicheva E., Chmielewski K., Stewart K.M., Ashall J., Everts B. (2015). Network integration of parallel metabolic and transcriptional data reveals metabolic modules that regulate macrophage polarization. Immunity.

[13] Ryan D.G., Murphy M.P., Frezza C., Prag H.A., Chouchani E.T., O’Neill L.A., Mills E.L. (2019). Coupling Krebs cycle metabolites to signalling in immunity and cancer. Nat. Metab..

[14] Van den Bossche J., O’Neill L.A., Menon D. (2017). Macrophage immunometabolism: Where are we (going)?. Trends Immunol..

[15] Covarrubias A.J., Aksoylar H.I., Yu J., Snyder N.W., Worth A.J., Iyer S.S., Wang J., Ben-Sahra I., Byles V., Polynne-Stapornkul T. (2016). Akt-mTORC1 signaling regulates Acly to integrate metabolic input to control of macrophage activation. Elife.

[16] Lauterbach M.A., Hanke J.E., Serefidou M., Mangan M.S.J., Kolbe C.C., Hess T., Rothe M., Kaiser R., Hoss F., Gehlen J. (2019). Toll-like receptor signaling rewires macrophage metabolism and promotes histone acetylation via atp-citrate lyase. Immunity.

[17] Van den Bossche J., van der Windt G.J.W. (2018). Fatty acid oxidation in macrophages and t cells: Time for reassessment?. Cell Metab..

[18] Vats D., Mukundan L., Odegaard J.I., Zhang L., Smith K.L., Morel C.R., Wagner R.A., Greaves D.R., Murray P.J., Chawla A. (2006). Oxidative metabolism and PGC-1 beta attenuate macrophage-mediated inflammation. Cell Metab..

[19] Divakaruni A.S., Hsieh W.Y., Minarrieta L., Duong T.N., Kim K.K.O., Desousa B.R., Andreyev A.Y., Bowman C.E., Caradonna K., Dranka B.P. (2018). Etomoxir inhibits macrophage polarization by disrupting coa homeostasis. Cell Metab..

[20] Nomura M., Liu J., Rovira I.I., Gonzalez-Hurtado E., Lee J., Wolfgang M.J., Finkel T. (2016). Fatty acid oxidation in macrophage polarization. Nat. Immunol..

[21] Namgaladze D., Zukunft S., Schnutgen F., Kurrle N., Fleming I., Fuhrmann D., Brune B. (2018). Polarization of human macrophages by Interleukin-4 does not require ATP-Citrate lyase. Front. Immunol..

[22] Baardman J., Verberk S.G.S., van der Velden S., Gijbels M.J.J., van Roomen C., Sluimer J.C., Broos J.Y., Griffith G.R., Prange K.H.M., van Weeghel M. (2020). Macrophage ATP citrate lyase deficiency stabilizes atherosclerotic plaques. Nat. Commun..

[23] Geeraerts X., Bolli E., Fendt S.M., Van Ginderachter J.A. (2017). Macrophage metabolism as therapeutic target for cancer, atherosclerosis, and obesity. Front. Immunol..

[24] Bonelli S., Geeraerts X., Bolli E., Keirsse J., Kiss M., Pombo Antunes A.R., Van Damme H., De Vlaminck K., Movahedi K., Laoui D. (2018). Beyond the M-CSF receptor—Novel therapeutic targets in tumor-associated macrophages. FEBS J..

[25] Zheng X., Turkowski K., Mora J., Brune B., Seeger W., Weigert A., Savai R. (2017). Redirecting tumor-associated macrophages to become tumoricidal effectors as a novel strategy for cancer therapy. Oncotarget.

[26] Hagemann T., Lawrence T., McNeish I., Charles K.A., Kulbe H., Thompson R.G., Robinson S.C., Balkwill F.R. (2008). “Re-educating” tumor-associated macrophages by targeting NF-kappaB. J. Exp. Med..

[27] Kiss M., Vande Walle L., Saavedra P.H.V., Lebegge E., Van Damme H., Murgaski A., Qian J., Ehling M., Pretto S., Bolli E. (2020). IL1beta promotes immune suppression in the tumor microenvironment independent of the inflammasome and gasdermin D. Cancer Immunol. Res..

[28] De Goede K.E., Driessen A.J.M., Van den Bossche J. (2020). Metabolic Cancer-Macrophage Crosstalk in the Tumor Microenvironment. Biology.

[29] Honkanen T.J., Tikkanen A., Karihtala P., Makinen M., Vayrynen J.P., Koivunen J.P. (2019). Prognostic and predictive role of tumour-associated macrophages in HER2 positive breast cancer. Sci. Rep..

[30] Zhao S., Torres A., Henry R.A., Trefely S., Wallace M., Lee J.V., Carrer A., Sengupta A., Campbell S.L., Kuo Y.M. (2016). ATP-Citrate lyase controls a glucose-to-acetate metabolic switch. Cell Rep..

[31] Remels L.M., De Baetselier P.C. (1987). Characterization of 3LL-tumor variants generated by in vitro macrophage-mediated selection. Int. J. Cancer.

[32] Movahedi K., Laoui D., Gysemans C., Baeten M., Stange G., Van den Bossche J., Mack M., Pipeleers D., In’t Veld P., De Baetselier P. (2010). Different tumor microenvironments contain functionally distinct subsets of macrophages derived from Ly6C(high) monocytes. Cancer Res..

[33] Schetters S.T.T., Rodriguez E., Kruijssen L.J.W., Crommentuijn M.H.W., Boon L., Van den Bossche J., Den Haan J.M.M., Van Kooyk Y. (2020). Monocyte-derived APCs are central to the response of PD1 checkpoint blockade and provide a therapeutic target for combination therapy. J. Immunother. Cancer.

[34] Baardman J., Verberk S.G.S., Prange K.H.M., van Weeghel M., van der Velden S., Ryan D.G., Wust R.C.I., Neele A.E., Speijer D., Denis S.W. (2018). A defective pentose phosphate pathway reduces inflammatory macrophage responses during hypercholesterolemia. Cell Rep..

[35] Zhou Y., Zhou B., Pache L., Chang M., Khodabakhshi A.H., Tanaseichuk O., Benner C., Chanda S.K. (2019). Metascape provides a biologist-oriented resource for the analysis of systems-level datasets. Nat. Commun..

[36] Van den Bossche J., Lamers W.H., Koehler E.S., Geuns J.M., Alhonen L., Uimari A., Pirnes-Karhu S., Van Overmeire E., Morias Y., Brys L. (2012). Pivotal advance: Arginase-1-independent polyamine production stimulates the expression of IL-4-induced alternatively activated macrophage markers while inhibiting LPS-induced expression of inflammatory genes. J. Leukoc. Biol..

[37] Van den Bossche J., Laoui D., Morias Y., Movahedi K., Raes G., De Baetselier P., Van Ginderachter J.A. (2012). Claudin-1, claudin-2 and claudin-11 genes differentially associate with distinct types of anti-inflammatory macrophages in vitro and with parasite- and tumour-elicited macrophages in vivo. Scand. J. Immunol..

[38] Langston P.K., Nambu A., Jung J., Shibata M., Aksoylar H.I., Lei J., Xu P., Doan M.T., Jiang H., MacArthur M.R. (2019). Glycerol phosphate shuttle enzyme GPD2 regulates macrophage inflammatory responses. Nat. Immunol..

[39] Rhee J., Solomon L.A., DeKoter R.P. (2019). A role for ATP Citrate Lyase in cell cycle regulation during myeloid differentiation. Blood Cells Mol. Dis..

[40] Gubin M.M., Esaulova E., Ward J.P., Malkova O.N., Runci D., Wong P., Noguchi T., Arthur C.D., Meng W., Alspach E. (2018). High-dimensional analysis delineates myeloid and lymphoid compartment remodeling during successful immune-checkpoint cancer therapy. Cell.

[41] Xue J., Schmidt S.V., Sander J., Draffehn A., Krebs W., Quester I., De Nardo D., Gohel T.D., Emde M., Schmidleithner L. (2014). Transcriptome-based network analysis reveals a spectrum model of human macrophage activation. Immunity.

[42] Benner B., Scarberry L., Suarez-Kelly L.P., Duggan M.C., Campbell A.R., Smith E., Lapurga G., Jiang K., Butchar J.P., Tridandapani S. (2019). Generation of monocyte-derived tumor-associated macrophages using tumor-conditioned media provides a novel method to study tumor-associated macrophages in vitro. J. Immunother. Cancer.

[43] Smith T.D., Tse M.J., Read E.L., Liu W.F. (2016). Regulation of macrophage polarization and plasticity by complex activation signals. Integr. Biol..

[44] Daley J.M., Brancato S.K., Thomay A.A., Reichner J.S., Albina J.E. (2010). The phenotype of murine wound macrophages. J. Leukoc. Biol..

[45] Azizi E., Carr A.J., Plitas G., Cornish A.E., Konopacki C., Prabhakaran S., Nainys J., Wu K., Kiseliovas V., Setty M. (2018). Single-Cell map of diverse immune phenotypes in the breast tumor microenvironment. Cell.

[46] Muller S., Kohanbash G., Liu S.J., Alvarado B., Carrera D., Bhaduri A., Watchmaker P.B., Yagnik G., Di Lullo E., Malatesta M. (2017). Single-cell profiling of human gliomas reveals macrophage ontogeny as a basis for regional differences in macrophage activation in the tumor microenvironment. Genome Biol..

[47] Munoz-Rojas A.R., Kelsey I., Pappalardo J.L., Chen M., Miller-Jensen K. (2021). Co-stimulation with opposing macrophage polarization cues leads to orthogonal secretion programs in individual cells. Nat. Commun..

[48] Ham S., Lima L.G., Lek E., Moller A. (2020). The impact of the cancer microenvironment on macrophage phenotypes. Front. Immunol..

[49] Guilliams M., Scott C.L. (2017). Does niche competition determine the origin of tissue-resident macrophages?. Nat. Rev. Immunol..

