# Supplementary material for: Myeloid-Specific Acly Deletion Alters Macrophage Phenotype In Vitro and In Vivo without Affecting Tumor Growth
Source: Cancers (Basel). 2021 Jun 19;13(12):3054. doi: 10.3390/cancers13123054 (PMC8235155; doi:10.3390/cancers13123054)
Supplement: Supplementary file 1 [file cancers-13-03054-s001.zip › cancers-1213534-supplementary.pdf]

Article

Myeloid-Specific Acly Deletion Alters Macrophage Phenotype *In Vitro* And *In Vivo* without Affecting Tumor Growth

Supplementary Files

Supplementary Table S1: List of antibodies used.

Target	Clone	Fluorochrome	Company
CD8a	53-6.7	BV421	Biolegend
CD4	RM4-5	BV510	Biolegend
Ly6C	HK1.4	BV605	Biolegend
CD11c	N418	BV650	Biolegend
CD64	X54-5/7.1	BV711	Biolegend
CD45	30-F11	BV785	Biolegend
Ly6G	1A8	FITC	Biolegend
MHCII	M5/114.15.2	PerCP-Cy5.5	Biolegend
CD19	6D5	PE	Biolegend
CD11b	M1/70	PE-Cy7	Biolegend
NK1.1	PK136	APC	Biolegend
CD3	17A2	AF700	Biolegend
Fixable Viability Dye	n/a	eFluor 780	eBioscience
IL-4Rα	mIL4R-M1	BV421	BD Biosciences
CD273	TY25	BV510	BD Biosciences
CD80	16-10A1	BV650	Biolegend
iNOS	CXNFT	APC	eBioscience
Arg1	A1exF5	PE	eBioscience
CD11c	N418	AF700	eBioscience
NK1.1	PK136	APC-Cy7	BD Biosciences
CD3	17A2	APC-Cy7	Biolegend
CD19	6D5	APC-Cy7	Biolegend
CD206	C068C2	BV421	Biolegend
CD40	3/23	BV510	BD Biosciences
CD43	S7	BV650	BD Biosciences
CD86	GL-1	APC	Biolegend
CD301	LOM-14	PE	Biolegend

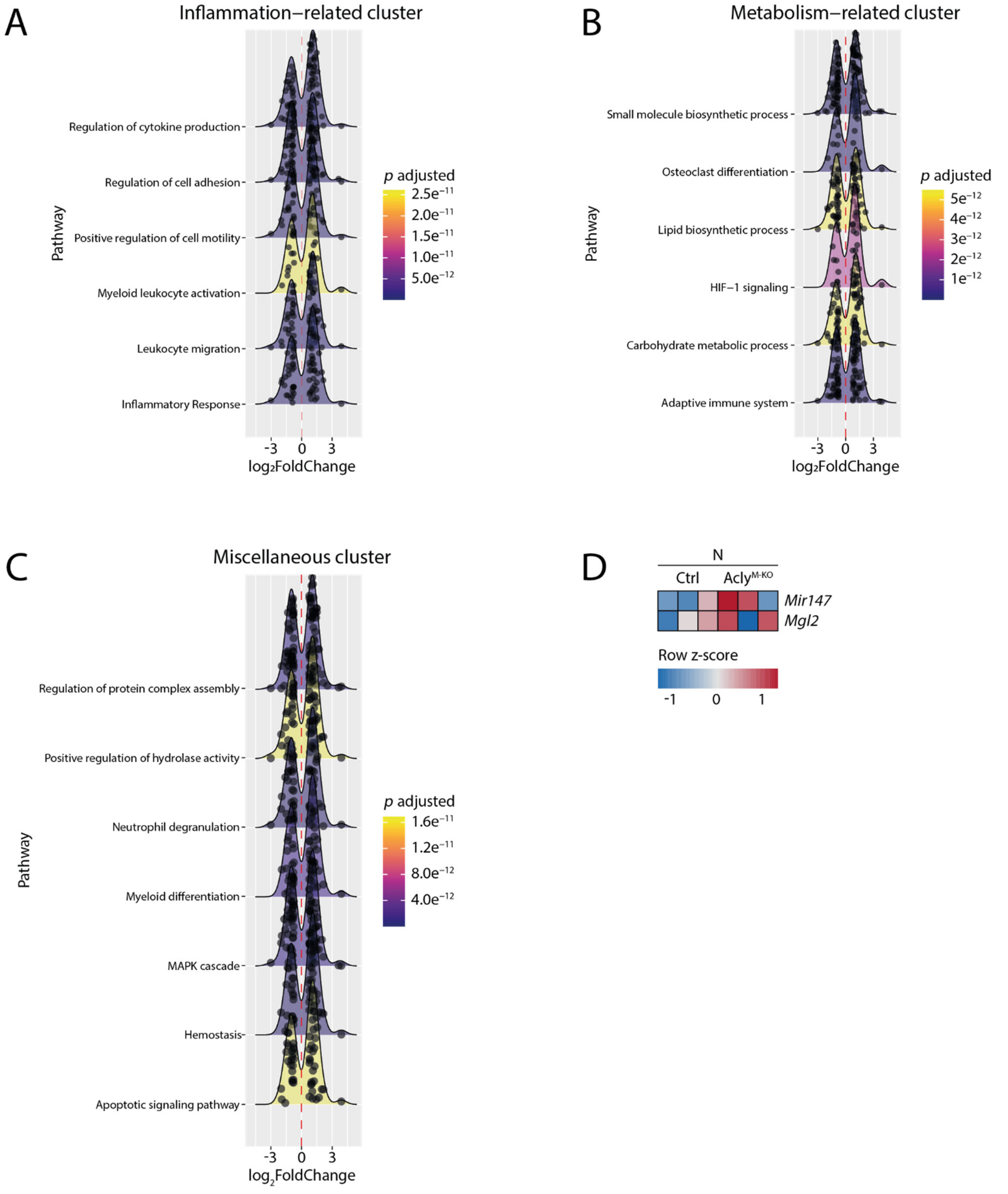

Supplementary Figure S1. Acly$^{M\text{-}KO}$ BMDMs do not show directionality in their IL-4-induced pathways and IL-4-induced genes are not affected at baseline. Ridgeline plots of differentially regulated pathways as identified in Figure 1B-D for the inflammation-related cluster (A), metabolism-related cluster (B) and miscellaneous cluster (C). Log2FoldChange of genes involved in the plotted pathways are shown on the x axis and color of the distributions represents pathway

adjusted p-value. (D) Significantly regulated IL-4-induced genes are not significantly regulated in unstimulated BMDMs.

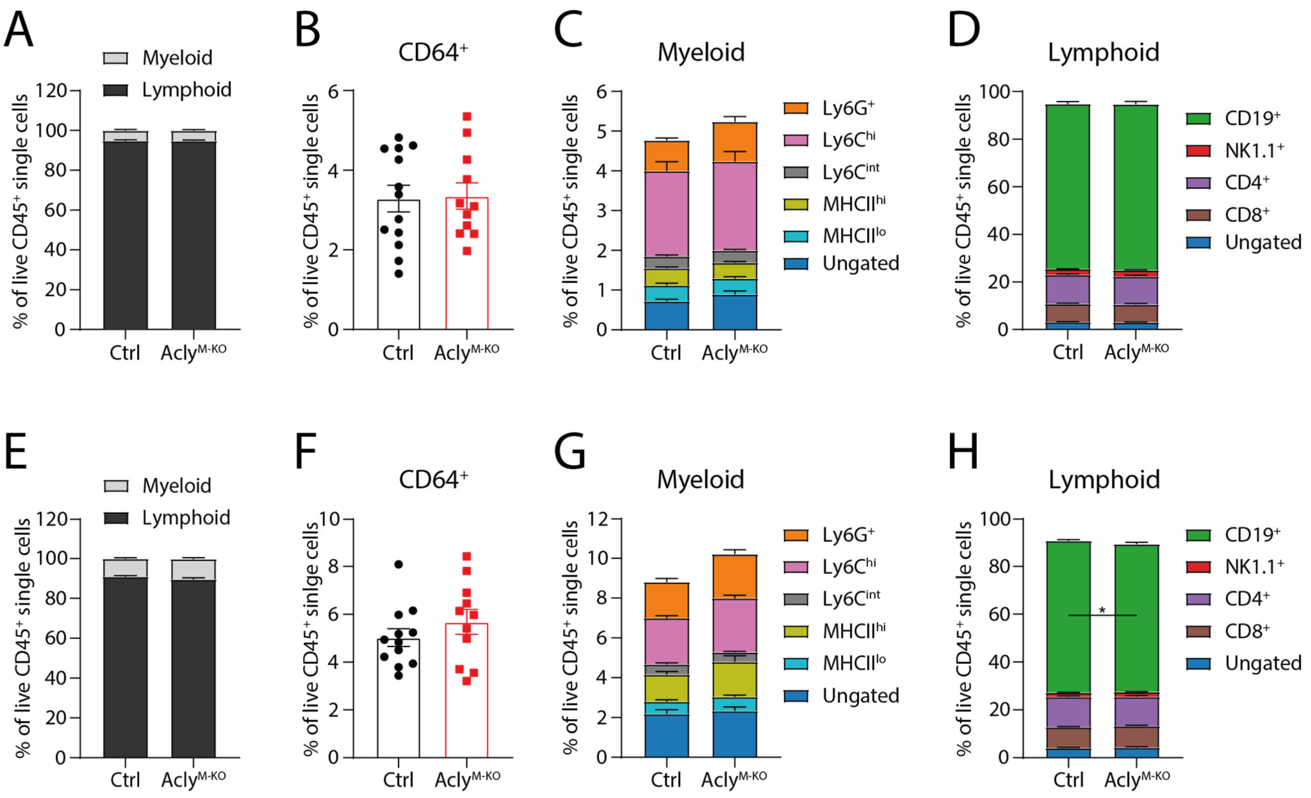

Supplementary Figure S2. Acly^{M-KO} does not affect splenic immune composition during *in vivo* tumor models. (A,E) Percentages of CD11b⁺ or CD11b⁻ cells of live CD45⁺ single cells. (B,F) Percentage of CD64⁺ of live CD45⁺ single cells. (C,G) Percentages of myeloid or (D,H) lymphoid cell populations of live CD45⁺ single cells. Figures A-D represent spleens of the MC38 tumor model while figures E-H represent spleens of the 3LLR tumor model. For C, D, G and H, colors match the colors used for manual gating in Figures 3E and 4E. P-values are indicated as <0.01**. Results are shown as mean ± SEM.

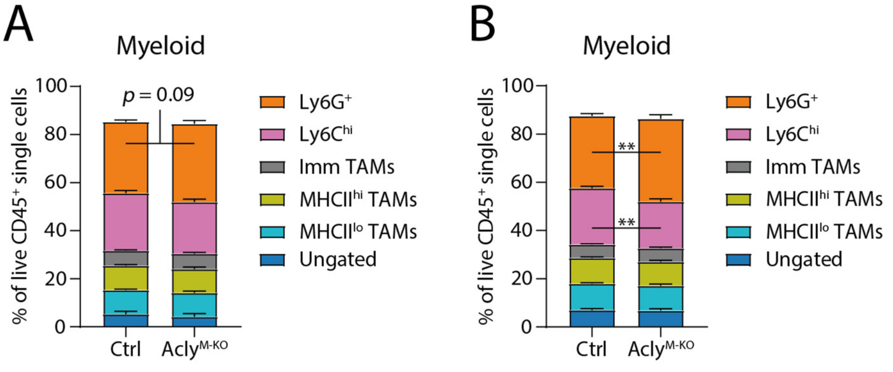

Supplementary Figure S3. Validation of myeloid subpopulation distribution in macrophage marker panels of 3LLR tumor model. Previously determined myeloid subpopulation distribution (Figure 4I) was validated in additional flow cytometry panels for macrophage phenotypic markers using the same backbone (Figure 5). (A) Percentages of myeloid cell populations of live CD45⁺ single cells of macrophage phenotypic panel 1 (Figure 5A-C, F). (B) Percentages of myeloid cell populations of live CD45⁺ single cells of macrophage phenotypic panel 2 (Figure 5D, E, G). Colors match the colors used for manual gating in Figure 4E. P-values are indicated as <0.01**. Results are shown as mean ± SEM.
